# Urinary Tract Infection among Symptomatic Outpatients Visiting a Tertiary Hospital Based in Midwestern Nigeria

**DOI:** 10.5539/gjhs.v5n2p187

**Published:** 2012-01-13

**Authors:** Otajevwo F. D.

**Affiliations:** 1 Department of Microbiology & Biotechnology, Western Delta University, Oghara, Nigeria

**Keywords:** urinary tract infection, symptomatic, outpatients, prevalence, Benin City

## Abstract

Microbial pathogens implicated in urinary tract infection and their antibiotic susceptibility patterns as prevalent in UTI symptomatic outpatients resident in Benin City, Nigeria was the focus of this study. One hundred (100) midstream urine samples were collected into sterile plastic universal bottles from outpatients who visited the University of Benin Teaching Hospital, Nigeria and who were tentatively diagnosed as manifesting symptoms of UTI. Patients were referred to the Medical Microbiology department by the consulting doctors. Significant bacterial counts and neutrophil (pus cells) counts were carried out on samples by standard methods. Positive samples for both counts were inoculated aseptically on sterile MacConkey agar, Cystine Lactose Electrolyte Deficient (CLED) agar and Sabouraud Dextrose agar plates and incubated appropriately. Microbial isolates were identified and antibiotic sensitivity testing was carried out on isolates by standard methods. Thirty nine (39.0%) and 61 (61.0%) samples recorded significant microbial growth and no growth respectively. Gram negative bacilli constituted 86.1% (of which enterobacteriaceae made up 49.9%) while gram positive cocci made up 13.9%. Strains of uropathogens isolated were *Alcaligenes* spp (19.4%), *Klebsiella aerogenes* (16.7%), *Escherichia coli* (13.9%), *Staphylococcus aureus* (13.9%), *Candida albicans* (11.1%), *Proteus mirabilis* (8.3%), *Pseudomonas aeruginosa* (5.5%), *Enterobacter* spp (5.5%) and *Providencia* spp (5.5%). Occurrence of UTI in male and female patients were 58.3% and 41.7% respectively of which UTI occurred highest in the 25-46, 15-54 and 27-54 age groups in that decreasing order. *Alcaligenes* spp occurred most in very old female patients. *Candida albicans* (the only fungal uropathogen) occurred in an 8day old male patient. Other isolates occurred in much older patients. A significantly high microscopic neutrophil count or pyuria was recorded from deposits of UTI positive patients (i.e. < 5/HPF). Eighteen (representing 50.5%) and 15 (47.8%) of total microbial strains isolated were sensitive to nitrofurantoin and ceftriaxone respectively. Antibiotic susceptibility profile also showed 13(41.6%), 13(41.6%), 13(41.6%) for ciprofloxacin, cefuroxime and ofloxacin respectively suggesting moderate sensitivity of the fluoroquinolones and second/third generation cephalosporins. Gentamicin, ampicillin and augmentin recorded over 70.0% resistance level each. A total of nineteen bacterial strains made of *E. coli, Enterobacter* spp, *Proteus mirabilis, Providencia* spp, *Staph. aureus* and *Pseudomonas aeruginosa* were multi drug resistant as they resisted 3, 3, 4, 4, 5 and 8 antibiotics respectively.

## 1. Introduction

Urinary tract infection is defined as the microbial invasion of any of the tissues of the urinary tract extending from the renal cortex to the urethral meatus (Kunin, 1979). The urinary tract includes the organs that collect and store urine and release it from the body and these organs include the kidneys, ureters, bladder, urethra and accessory structures. Urine formed in the kidney is a sterile fluid that serves as a good culture medium for the proliferation of bacteria (Omonigho et al., 2001).

UTI is one of the most common infectious diseases which has been extremely studied in the field of clinical practice (Dulawa, 2003). It is the most common health care – associated group of bacterial infections affecting humans in Africa (Ozumba, 2005). UTI is among the most common bacterial infections in humans both in community and hospital settings which occurs in all age groups and in both genders (Orret & Davis, 2006; Omoregie et al., 2008). UTI is the major cause of morbidity in both the hospital and community settings (Omigie et al., 2009) and affecting both out and in patients (Suwangool, 2012).

The evidence of UTI is confirmed by the presence of 105 microorganisms or of a single strain of bacterium per milliliter in two consecutive midstream samples of urine (Berg, 1985; Davidson et al., 1989). UTI could be described based on the part of the tract affected. For the upper tract it is called pyelonephritis and for the lower part, cystiis (Stamm, 1998). As an anatomical unit, an infection of any part can generally spread to its other parts (Behzadi, 2008; Howes, 2010; Cunha, 2010; Kolawole et al., 2009).

The commonest mode of infection is the ascending route through which organisms of the bowel flora contaminate the urethra, ascend to the bladder and migrate to the kidney or prostate. Haematogenous spread do occur particularly during neonatal period (Azubuike et al., 1999).

Despite the presence of several antibacterial factors in urine such as pH, urea concentration, osmolarity, various organic acids, salt content of the urine, urinary inhibitors to bacterial adherence (such as Tamm-Horsfall protein or THP, bladder mucopolysaccharide, low molecular weight oligosaccharide, secretory IgA and lactoferrin), the uropathogenic bacteria are able to adhere, grow and resist against host defenses which finally result in colonization and infection of the urinary tract (Cunha, 2010; Dulawa, 2003; Hooton et al., 1996).

Microbiologically, urinary tract infection exists when pathogenic microorganisms are detected in the urinary tract (Travis & Bruhard, 1991; Tolkoff & Rubin, 1986). The infection is considered significant and requires treatment when more than 105 microorganisms (CFU) per milliliter of urine are present in a properly collected specimen (Travis & Bruhard, 1991; Tolkoff & Rubin, 1986). Gram negative bacteria such as *Escherichia coli*, *Proteus* spp, *Klebsiella* spp, *Enterobacter* spp, *Serratia* spp and *Pseudomonas* spp are usually detected in recurrent infections especially in association with stones, obstruction, urologic manipulation and nosocomial catheter-associated infections (Naylor, 1984; Travis & Bruhard, 1991; Warren, 1987). Microbial sensitivity tests should be done first in order to direct therapy of the urinary tract infection (Kunin, 1985).

Gram negative bacteria have been found most frequently in UTI cases by several authors with *Escherichia coli* and *Klebsiella* spp being the most predominant organisms (Ayan et al., 1988; DeMouy et al., 1988; Eghafona et al., 1988; Omonigho et al., 2001; Ebie et al., 2001). Other bacterial pathogens frequently isolated include *Staph aureus*, *Staph. epidermidis* and *Strept. faecalis* (Eghafona et al., 1988; Omonigho et al., 2001). For many years, pathogens associated with uncomplicated UTI have remained constant with *E.coli* identified as aetiologic agent in about 75-90% of UTIs (Karlowsky, 2002; Nakhjavani et al., 2007; Omigie et al., 2009). The remaining gram negative urinary pathogens are *Klebsiella* spp, *Proteus mirabilis* and *Pseudomonas aeruginosa*. *Enterococci* and coagulase negative Staphylococci e.g *Staph saprophyticus* are the most frequently implicated gram positive organisms (Shankel, 2007).

The emergence of antibiotic resistance in the management of urinary tract infections is a serious public health problem particularly in the developing World where apart from high level of poverty, ignorance and poor hygiene practices, there is also a high prevalence of fake and spurious drugs of questionable quality in circulation (Abubakar, 2009). Hence, the changing spectrum of microorganisms involved in urinary tract infections and emergence of resistance across institutions and geographical areas have made imperative the conduct of antibiotic susceptibility testing study of UTI pathogens in various regions from time to time.

It is to extend the frontiers of available information in this area that this study aimed at investigating urinary tract infection among symptomatic outpatients visiting a tertiary hospital based in western Nigeria was carried out with the following objectives: 1. determine the frequency distribution of microbial pathogens in UTI cases of outpatients resident in Benin City urban metropolis, 2. determine the sex distribution of microbial pathogens in UTI cases of outpatients in the study area, 3. determine the age and sex distribution of uropathogens in relation to significant neutrophil counts in UTI cases of outpatients in the study area, 4. determine sensitivity profile of uropathogens to selected antibiotics, 5. determine the multi drug resistance spread of uropathogens implicated in UTI cases of present study.

## 2. Materials and Methods

### 2.1 Population Study

A total of one hundred midstream urine samples were collected into sterile screw-capped universal containers containing few crystals of boric acid as preservative from outpatients who had visited the University of Benin Teaching Hospital, Edo State, Nigeria to see doctors with various complaints which were diagnosed tentatively as symptoms of urinary tract infection (UTI). The consulting doctors had then referred the patients to the Medical Microbiology laboratory for urine mcs (microscopy, culture and sensitivity) investigation for the purpose of making definite diagnosis. Recruited outpatients were instructed on how to collect the samples. All collected samples were appropriately labeled and processed immediately. Study was carried out between February, 2012 and July, 2012.

### 2.2 Sample Processing

#### 2.2.1 Test for Significant Bacterial Count

All urine samples were tested for significant bacteriuria by use of a modified semi-quantitative technique described by Mbata (2007). A standard bacteriological loopful of each urine sample (0.01ml) was spread over the surface of sterile Cystine Lactose Electrolyte Deficient (CLED) agar plates (LabM, UK). After inoculation, the plates were left on the bench for sometime in order to allow the urine to be absorbed into the agar medium. The plates were then inverted and incubated at 37°C for 18-24 hours. The number of bacterial colonies were counted and multiplied by 100 to give an estimate of the number of bacteria per milliliter of urine. A significant bacterial count was taken as any count equal to or in excess of 105 per milliliter.

#### 2.2.2 Confirmation of Significant Bacterial Count by Microscopy

All samples that recorded significant bacterial counts were subjected to urine microscopy test to detect presence of five pus cells per high power focus using X40 objective microscopically. All samples that were positive for significant bacterial count and also recorded five pus cells and above were then cultured on laboratory media.

#### 2.2.3 Cultural Studies

Positive samples for both significant bacteriuria and pus cells tests were cultured aseptically on sterile Sabouraud Dextrose agar (LabM, UK), MacConkey agar (LabM, UK), CLED agar (LabM, UK), and Blood agar plates according to standard methods. All inoculated plates were incubated at 37°C for 24hours. Pure isolates were then obtained and identified according to schemes provided by Alexopoulos (1996) and Cowan and Steel (1993). All identified isolates were subjected to sensitivity testing. Fungal isolates were excluded from antibiotic sensitivity testing.

#### 2.2.4 Antibiotic Sensitivity Testing

The agar diffusion disc technique described by Bauer et al. (1996) was applied. A colony of each pure isolate was streaked on sterile Nutrient agar plates aseptically using sterile inoculating wire loop. The appropriate multi-discs containing minimum inhibitory concentrations (MIC) of ciprofloxacin (10ug), ampicillin (30ug),

Nitrofurantoin (300ug), ceftriaxone (30ug), gentamicin (10ug), cefuroxime (30ug), ofloxacin (10ug), cefixime (10ug), ceftazidime (30ug) and augmentin (30ug) were then aseptically placed (impregnated) firmly onto the surface of the dried plates using sterile forceps.

The plates were left at room temperature for one hour to allow diffusion of the different antibiotics from the disc into the medium. The plates were then incubated at 37°C for 18-24hours. Interpretation of results was done using the length of inhibition of zone diameter. Zones of inhibition greater than 10mm were considered sensitive, 5-10mm moderate sensitive and no zone of inhibition, resistant (NCCLS, 2000).

## 3. Results

Table 1 shows the uropathogens that were isolated and identified. Gram negative, raised, motile, haemolytic, oxidase positive, citrate positive, urease negative and indole negative bacilli strains were identified as *Alcaligenes* spp. Gram negative, mucoid, non-motile, lactose, adonitol, inositol, glucose fermenting, voges praskauer positive, urease positive, citrate positive and indole negative bacilli strains were identified as *Klebsiella aerogenes*. All gram negative, raised, entire, circular, motile, lactose, glucose fermenting, indole positive, methyl red positive, voges praskauer negative, citrate negative and urease negative bacilli strains were identified as *Escherichia coli*. Bacterial strains that were gram positive cocci in clusters, catalase positive, DNAase negative, mannitol fermenting, raised, round and smooth colonies were identified as *Staphylococcus aureus*. Gram positive yeast cells, pseudohyphae positive, chlamydospores positive, germ tube positive, glucose, maltose, galactose and sucrose assimilation positive colonies were identified as *Candida albicans*

**Table 1:** Frequency distribution of microbial pathogens in midstream urine of outpatients studied

Microbial Isolates/Strains	No *of strains*	Frequency%
*Alcaligens* spp	7	19.4
*Klebsiella aerogenes*	6	16.7
*Escherichia coli*	5	13.9
*Staphylococcus aureus*	5	13.9
*Candida albicans*	4	11.1
*Proteus mirabilis*	3	8.3
*Pseudomonas aeruginosa*	2	5.5
*Enterobacter* spp	2	5.5
*Providencia* spp	2	5.5
**Total (9)**	**36**	**100.0%**

Gram negative bacteria: 86.1%

Gram positive bacteria: 13.9%

Enterobacteriaceae: 49.9%

Non-Enterobacteriaceae: 50.1%

Gram negative, swarming, fish odour colonies on sodium chloride-containing media, indole negative and urease positive strains were identified as *Proteus mirabilis*. Large, flat, opaque, aerobic, irregular colonies having grape-like smell, yellow-green pyocyannin pigment producing colonies on common culture media, oxidase positive colonies which grow at 42°C were identified as *Pseudomonas aeruginosa*. Strains and colonies that were gram negative, motile, non-sporing, lactose fermenting, indole negative, methyl red negative, voges praskaeur positive and citrate positive were confirmed to be *Enterobacter* spp. Gram negative, motile, non-sporing, non-lactose fermenting, methyl red positive, voges praskauer negative and phenylalanine deaminase positive colonies were identified as *Providencia* spp strains.

The frequency of occurrence of the isolates and their strains therefore, were as follows: *Alcaligenes* spp (19.4%), *Klebsiella aerogenes* (16.7%), *Escherichia coli* (13.9%), *Staphylococcus aureus* (13.9%), *Candida albicans* (11.1%), *Proteus mirabilis* (8.3%), *Pseudomonas aeruginosa* (5.5%), *Enterobacter* spp (5.5%), and *Providencia* spp (5.5%) in that descending order.

In Table 2, the sex distribution of the strains of the uropathogens is shown. Out of the total 36 strains isolated, 21(58.3%) and 15(41.7%) strains were isolated from male and female outpatients respectively. The highest occurring pathogens in male patients were *Klebsiella aerogenes* (23.8%), *Alcaligens* spp (19.1%) and *Staphylococcus aureus* (19.1%). In the female patients, the highest occurring pathogens were *Alcaligens* spp (20.0%), *Escherichia coli* (20.0%) and *Candida albicans* (20.0%). Whereas the least occurring uropathogens in male patients were *Candida albicans* (4.8%) and *Providencia* spp (4.8%), *Klebsiella aerogenes* (6.7%), *Staphylococcus aureus* (6.7%) and *Providencia* spp (6.7%) were the least occurring pathogens in female outpatients. *Pseudomonas aeruginosa* and *Enterobacter* spp were not isolated at all from male and female patients respectively

**Table 2:** Sex distribution of uropathogens in midstream urine of outpatients studied

Microbial Isolates/strains	No of strains	Males (%)	No of strains	Females (%)	Total
*Alcaligens* spp	4	19.1	3	20.0	7
*Klebsiella aerogenes*	5	23.8	1	6.7	6
*Escherichia coli*	2	9.5	3	20.0	5
*Staphylococcus aureus*	4	19.1	1	6.7	5
*Candida albicans*	1	4.8	3	20.0	4
*Proteus mirabilis*	2	9.5	1	6.7	3
*Pseudomonas aeruginosa*	0	0.0	2	13.3	2
*Enterobacter* spp	2	9.5	0	0.0	2
*Providencia* spp	1	4.8	1	6.7	2
**Total (9)**	**21(58.3%)**		**15(41.7%)**		**36(100.0%)**

The age and sex distribution of pathogens in relation to significant neutrophil (pus cells) count is shown in Table 3. In male outpatients, *Alcaligens* spp, *Proteus mirabilis, Klebsiella aerogenes* and *Staphylococcus aureus* were were isolated from 15-54years (average 38yrs), 22-44years (33yrs average), 25-46years (34yrs average), and 27-54years (44yrs average) age brackets respectively. These groups consisted of teenager, adolescent and young male outpatients. Of note, is *Candida albicans* which was isolated from an 8day old male outpatient. *Escherichia coli*, *Enterobacter* spp and *Providencia* spp were obtained from much older male outpatients. On the whole, the average age bracket of the male outpatients sampled was 8 days-52 years. *Klebsiella aerogenes, Pseudomonas aeruginosa, Candida albicans* and *Alcaligens* spp were isolated from 15-33years (24years average), 18-47years (33yrs average), 20-30years (32yrs average) and 25-83years (53yrs average) female outpatient age groups respectively. As in the male patients, these four female age groups consisted of adolescent and young female outpatients with exception of the 25-83year group where some very old female patients were recorded to be infected with *Alcaligens* spp.

**Table 3:** The age and sex distribution of microbial pathogens in relation to significant neutrophil (pus cells) counts in urine samples of patients

Microbial Isolates/strains	Sex of Patients	Age Bracket of Patients showing sig cultural Yield (yrs)	Average age of Patients showing sig cultural yield (yrs)	Average neutrophil count/HPF
*Alcaligens* spp	M (4)	15-54	38	21
	F (3)	25-83	53	NA
*Klebsiella aerogenes*	M (5)	25-46	34	19
	F (1)	15-33	24	NA
*Escherichia coli*	M (2)	36-78	52	16
	F (3)	40-52	46	NA
*Staphylococcus aureus*	M (4)	27-54	44	10
	F (1)	33-50	42	NA
*Candida albicans*	M (1)	8days	8days	23
	F (3)	20-30	32	2
*Proteus mirabilis*	M (2)	22-44	33	9
	F (1)	30-52	41	NA
*Pseudomonas aeruginosa*	M (0)	NA	NA	6
	F (2)	18-47	33	NA
*Enterobacter* spp	M (2)	36-57	47	7
	F (0)	NA	NA	NA
*Providencia* spp	M (1)	30	30	6
	F (1)	46	46	NA
Total:	M (21)			
	F (15)			

NA, Not Available

HPF, High Power Focus

*Escherichia coli, Staphylococcus aureus, Proteus mirabilis*, and *Providencia* spp were isolated from much older female outpatients. Of note, is *Escherichia coli* and *Alcaligens* spp which were isolated from very old female patients. The average age bracket of the female outpatients was 24-53years.

The highest pus cell count (neutrophil count) of 23/HPF was obtained from the midstream urine deposit of the 8day old male patient from whom *Candida albicans* (a fungal uropathogen) was isolated. Pus cell counts of 21/HPF, 19/HPF, 16/HPF and 10/HPF were obtained microscopically from urine deposits of outpatients and from which *Alcaligens* spp, *Klebsiella aerogenes*, *Escherichia coli* and *Staphylococcus aureus* uropathogens were isolated respectively. The least count of 6/HPF was obtained each for *Pseudomonas aeruginosa* and *Providencia* spp.

The antibiotic sensitivity profile of the 36 uropathogens to ciprofloxacin, ampicillin, nitrofurantoin, ceftriaxone, gentamicin, cefuroxime, ofloxacin, cefixime, ceftazidime and augmentin is shown in Table 4. Three (42.9%), 2 (33.3%), 1 (20.0%), 1 (20.0%), 1 (20.0%), 1 (20.0%) and 1 (20.0%) strains of *Alcaligens* spp, *Klebsiella aerogenes, Escherichia coli, Staphylococcus aureus, Proteus mirabilis, Enterobacter* spp and *Providencia* spp respectively were sensitive to all the ten antibiotics on the average. No *Pseudomonas aeruginosa* strain was sensitive on average. A higher percentage of pathogens resisted all ten antibiotics compared to those that were sensitive.

**Table 4:** Susceptibility profile of uropathogenic bacterial organisms to selected antibiotics after 24 hours incubation at 37°C

Microbial Isolates/strains	SELECTED ANTIBIOTICS USED																					
	CIP(%)		PN(%)		NIT(%)		CRO(%)R		GEN(%)		CRX(%)		OFX(%)		CXM(%)		CAZ(%)		AUG(%)		MEANS	
	S	R	S	R	S	R	S	R	S	R	S	R	S	R	S	R	S	R	S	R	S	R
*Alcaligens spp*	3	4	3	4	4	3	6	1	3	4	4	3	4	3	1	6	1	6	1	6	3	4
*n = 7(19.4%)*	42.9	57.1	42.9	57.1	57.1	42.9	85.7	14.3	42.9	57.1	57.1	42.9	57.1	42.9	14.3	85.7	14.3	85.7	14.3	85.7	42.9	57.1
*Klebsiella aerogenes*	2	4	2	4	2	4	1	5	1	5	2	4	2	4	2	4	0	6	1	5	2	4
*n =6(16.7%)*	33.3	66.7	33.3	66.7	33.3	66.7	16.7	83.3	16.7	83.3	33.3	66.7	33.3	66.7	33.3	66.7	0.0	100.0	16.7	83.3	33.3	66.7
*Escherichia coli*	1	4	1	4	4	1	1	4	1	4	1	4	1	4	0	5	0	5	0	5	1	4
*n = 5(13.9%)*	20.0	80.0	20.0	80.0	80.0	20.0	20.0	80.0	20.0	80.0	20.0	80.0	20.0	80.0	0.0	100.0	0.0	100.0	0.0	100.0	20.0	80.0
*Staphylococcus aureus*	1	4	0	5	5	0	3	2	0	5	2	3	0	5	1	4	0	5	0	5	1	4
*n = 5(13.9%)*	20.0	80.0	0.0	100.0	100.0	0.0	60.0	40.0	0.0	100.0	40.0	60.0	0.0	100.0	20.0	80.0	0.0	100.0	0.0	100.0	20.0	80.0
*Candida albicans*	**DOES NOT RESPOND TO BACTERIAL ANTIBIOTICS**																					
*n = 4(11.1%)*																						
*Proteus mirabilis*	2	1	1	2	1	2	0	3	1	2	1	2	2	1	0	3	0	3	0	3	1	2
*n = 3(8.3%)*	66.7	33.3	33.3	66.7	33.3	66.7	0.0	100.0	33.3	66.7	33.3	66.7	66.7	33.3	0.0	100.0	0.0	100.0	0.0	100.0	33.3	66.7
*Pseudomonas aeruginosa*	0	2	0	2	0	2	1	1	0	2	1	1	1	1	0	2	0	2	0	2	0	2
*n = 2(5.5%)*	0.0	100.0	0.0	100.0	0.0	100.0	50.0	50.0	0.0	100.0	50.0	50.0	50.0	50.0	0.0	100.0	0.0	100.0	0.0	100.0	0.0	100.0
*Enterobacter spp*	2	0	2	0	1	1	1	1	1	1	1	1	2	0	0	2	0	2	0	2	1	1
*n = 2(5.5%)*	100.0	0.0	100.0	0.0	50.0	50.0	50.0	50.0	50.0	50.0	50.0	50.0	100.0	0.0	0.0	100.0	0.0	100.0	0.0	100.0	50.0	50.0
*Providencia spp*	1	1	0	2	1	1	2	0	0	2	1	1	1	1	1	1	0	2	0	2	1	1
*n = 2(5.5%)*	50.0	50.0	0.0	100.0	50.0	50.0	100.0	0.0	0.0	100.0	50.0	50.0	50.0	50.0	50.0	50.0	0.0	100.0	0.0	100.0	50.0	50.0
TOTAL	13	19	9	23	18	14	15	17	7	25	13	19	13	19	5	27	1	31	2	30		
n = 36(100.0%)	41.6	58.4	28.7	71.3	50.5	49.5	47.8	52.2	20.4	79.6	41.6	58.4	41.6	58.4	14.7	85.3	14.3	85.7	22.7	77.3		

In terms of effectiveness of each antibiotic, 18 (50.5%), 15 (47.8%), 13 (41.6%) and 13 (41.6%) strains of all isolated pathogens were sensitive to nitrofurantoin, ceftriaxone (rocephine), ciprofloxacin, cefuroxime and ofloxacin (tarivid) respectively. Nine (28.7%), 7 (20.4%) and 5 (14.7%) strains of all pathogens were sensitive to ampicillin, gentamicin and cefixime respectively. In decreasing order, 31 (85.7%), 30 (77.3%), 27 (85.3%), 25 (79.6%) and 23 (71.3%) of strains were resistant to ceftazidime, augmentin, cefixime, gentamicin and ampicillin

Whereas *E.coli* and *Enterobacter* spp resisted three antibiotics each, *Proteus mirabilis* and *Providencia* spp were resistant to four drugs each while *Staph. aureus* and *Pseudomonas aeruginosa* were resistant to five and above six antibiotics respectively in this study (Table 5).

**Table 5:** Multidrug resistance occurrence of uropathogens in UTI cases of outpatients in study area

**NO OF ANTIBIOTICS RESISTED**				
Bacterial Isolates/strains	3 drugs	4 drugs	5 drugs	Above 6 drugs
*Escherichia coli* (n = 5)	+	-	-	-
*Enterobacter* spp (n = 2)	+	-	-	-
*Proteus mirabilis* (n = 3)	-	+	-	-
*Providencia* spp (n = 2)	-	+	-	-
*Staph aureus* (n = 5)	-	-	+	-
*Pseud. aeruginosa* (n = 2)	-	-	-	+

## 4. Discussion

This study was an attempt to determine the microbial pathogens implicated in urinary tract infection and their antibiotic susceptibility patterns as prevalent in UTI symptomatic outpatients resident in the metropolitan (urban) area of Benin City, the capital of former Midwestern region of Nigeria. Out of the 100 processed samples, 39 (39.0%) midstream urine samples yielded significant microbial growth. Sixty one (61.0%) samples recorded no bacterial and fungal growth after 24-48hours incubation at 37°C. The reason for this may be due to the fact that subjects (from whom negative samples were obtained), may have been on antibiotic therapy before reporting to the hospital and laboratory. The antibiotics may have inhibited bacterial growth (Okonofua, 1989). The use of midstream urine was aimed at reducing and eliminating the influence of normal flora and other contaminants on expected results.

In this study, a UTI prevalence rate of 39.0% was obtained. This is consistent with the reports of similar UTI studies by other workers (Ebie et al., 2001; Oladeinde et al., 2011) who recorded 35.3% and 39.7% respectively. Other studies recorded much lower prevalence rates of 26.7% (Sobozak et al., 1999) and 22.3% (Omonigho et al., 2001). It is on record that much higher prevalence rates have been reported by some authors (Shirishkumar et al., 2012; Obiogbolu et al., 2009; Onifade et al., 2005; Elder et al., 1971; Jellheden et al., 1996) stating prevalence rates of 46.5%, 54.0%, 58.0%, 66.0% and 71.6% respectively.

Results obtained in this study also showed that gram negative bacilli constituted 86.1% (of which enterobacteriaceae made up 49.9%) while gram positive cocci which constituted 13.9% of which the most prevalent and second most prevalent bacterial agents of UTI in the study area were *Alcaligens* spp (19.4%) and *Klebsiella aerogenes* (16.7%) respectively. This is similar to the report of Oluremi et al. (2011) which stated 85.0% of gram negative bacilli (made of 66.7% enterobacteriaceae) and 15.0% gram positive cocci of which *Escherichia coli* and *Klebsiella* spp were the most and second most occurring bacterial agents respectively. The present report also agrees with reports of other studies (Grunerberg, 1980; Orret and Shurland, 1998; Daza et al., 2001; Dimitrov et al., 2004; Inabo and Obanibi, 2006; Dash, 2008; Abubakar, 2009; Omigie, 2009; Nwadioha et al., 2010) but differs from the reports of Ehinmidu (2003) and Aboderin et al. (2009) which recorded *Pseudomonas aeruginosa* and *Klebsiella* spp respectively as the predominant bacteria.

In this study, other bacterial pathogens isolated were *Escherichia coli* (13.9%), *Staphylococcus aureus* (13.9%), *Proteus mirabilis* (8.3%), *Pseudomonas aeruginosa* (5.5%), *Enterobacter* spp (5.5%) and *Providencia* spp (5.5%).

The isolation of *Alcaligens* spp in this study was of much interest. According to Brooks et al., (2004), *Alcaligens* spp normally occurs as part of normal human bacterial flora but has been isolated from respirators, renal dialysis systems etc as an opportunistic pathogen. The focus here is on dialysis system as it relates to old patients from whom it was isolated. The isolation of *Klebsiella aerogenes* as the second most occurring pathogen of UTI in the present study environment is consistent with reports of other studies (Gales et al., 2000; Al-Sweih et al., 2005; Uwaezuoke & Ogbulie, 2006; Haghl-Astelani et al., 2007; Dash, 2008; Abubakar, 2009; Obiogbolu et al., 2009; Mbata, 2007).

There has been disparity in findings of previous researchers as to which of *Escherichia coli* and/or *Klebsiella* spp is/are predominant in UTI cases. While some workers reported *E.coli* as most occurring (Obiogbolu et al., 2009; Oluremi et al., 2011; Ayhan et al., 1988; Akinyemi et al., 1997; Okonofua & Okonofua, 1989; De-Mouy et al., 1988; Eghafona et al., 1988; Delzell, 2000; Ebie et al., 2001; Onifade et al., 2005; Aiyegoro et al., 2007; Mbata, 2007; Oladeinde et al., 2011), other workers maintained that *Klebsiella* spp was the most implicated (Omonigho et al., 2001; Nwadioha et al., 2010; Habib, 2012; Mazokopakis & Potolidis, 2012), other workers maintained that *Klebsiella* spp was the most implicated (Omonigho et al., 2001; Nwadioha et al., 2010). This study, however, further confirmed the prominent involvement of both bacterial pathogens in UTI cases as earlier established by Logoria and Gonzalez (1987) and Obiogbolu et al. (2009).

The low occurrence of *Pseudomonas aeruginosa* (5.5%) in this study is not in agreement the much higher incidence rates recorded by other authors in similar UTI investigations (Oluremi et al., 2011; Orret & Shurland, 1988; Shigemura et al., 2005; Tambekar et al., 2006; Kolawole, 2009; Omigie et al., 2009).

*Staphylococcus aureus* occurred more in male outpatients (19.1%) than in female outpatients (6.7%) Table 2. The reason for this, is not clear but lack of circumcision, receptive anal intercourse (as in homosexuals) and HIV infection may predispose males to UTI (Orret et al., 2006).

The high occurrence of enterobacteriaceae in this work (86.1%) and some of the isolated pathogens being coliforms show that a high percentage of urinary tract infection may be due to faecal contamination arising from poor hygiene. Also, coliform organisms are indicators of poor safety habits, poor hygiene and poor sanitary lifestyle (Behzadi et al., 2008). This is so because, these organisms occur in the perineum of the large intestine as commensals (Behzadi et al., 2008; Moore et al., 2002; Anyamene et al., 2002). Also, commensals of the intestine are more involved in UTIs because of the anatomy proximity to the genitor-urinary area (Obiogbolu, 2004).

Observed data in this study also showed a higher UTI prevalence in males (58.3%) than in the female outpatients (41.7%) (Table 2). The reason for this was not clear as it contradicts reports of earlier studies on UTI (Orret and Shurland, 1999; Gales et al., 2000; Tambekar et al., 2006; Mbata, 2007; Adedeji & Abdulkadir, 2009; Kebira et al., 2009; Kolawole, 2009; Oladeinde et al., 2011).

UTI in this study occurred highest in the 25-46year (34yr average) age group followed by 15-54year (38yr average) and 27-54year (44yr average) age group. These age brackets consist of teenagers, adolescents and young people. This may be due to increased sexual activity which predisposes to UTI and is supported by reports of similar studies on UTI by earlier authors (Oluremi et al., 2011; Oladeinde et al., 2011). Finding is also consistent with reports of Kebira et al. (2009) and Kolantar et al. (2008) which reported higher male to female ratio among neonates and young children but not in agreement with reports of other workers (Mbata, 2007; Akinyemi et al., 1999; Ibeawuchi & Mbata, 2002). The occurrence of low UTI in patients above 54 years (Table 3) agrees with data of Omigie et al. (2009) and Dimitrov et al. (2004) and is incongruous with reports of Oluremi et al. (2011), Shigemura et al. (2005), Oladeinde et al. (2011) and McCue (2012).

Microbial pathogens distribution with respect to age groups as presented in Table 3 shows that *Alcaligens* spp, *Proteus mirabilis*, *Klebsiella aerogenes* and *Staphylococcus aureus* were isolated from outpatients of 38years, 33 years, 34 years and 44 years (average age) respectively. Although no statistics was done to ascertain any significant association of these organisms with young outpatients, it seems to ordinarily suggest so. The occurrence however, of *Alcaligens* spp in very old female patients seems to strike attention in terms of eliciting further investigation. It may however, be suggestive of emergence of UTI due to this pathogen as a complication of dialysis since the pathogen has been implicated in dialysis systems (Brooks et al., 2004). Dialysis is a long standing recommended therapy for renal (kidney) disease or failure and old people are more prone to renal disease (Brooks et al., 2004). No reason could however be adduced to the occurrence of *Alcaligens* spp in old female outpatients only as shown in this study. An extensive investigation into the occurrence of this agent in UTI in old people particularly those on dialysis is hereby recommended.

*Escherichia coli, Staphylococcus aureus, Proteus mirabilis, Providencia* spp and *Enterobacter* spp seemed to have caused UTI in much older patients. Again, since no statistics was done, a definite conclusion could not be made as this may have occurred by chance. Of note also, was the occurrence of *Candida albicans* (the only fungal uropathogen in this study) in an 8day old male outpatient. The neutrophil (pus cells) count of 23/HPF obtained from the microscopic view of the urine deposit of this neonate baby indicated an acute on going infection. While it is possible that the high count may be due to immunological response to other underlining systemic disorders in the child, the isolation of significant growth of *Candid albicans* is indeed alarming. Although the reason for this was not immediately known, there is a possibility of congenital transmission from a mother who may have had acute systemic candidiasis. This agrees with the report of Kebira et al. (2009) which stated that rates of UTI in boys is associated with high incidence of congenital malformations and uncircumcised genitalia that often gets contaminated from the prepuce or introtal area that is not always clean. Significant pyuria was obtained for the other isolates though in varying degrees (Table 3).

Isolation of pathogens from any diseased human body site alone may be of no use without a corresponding prescription of appropriate antibiotic therapy. Besides, the need for constant monitoring of susceptibility of specific pathogens in different populations to commonly used antimicrobial agents has been suggested (Shirishkumar et al., 2012). For this purpose, antibiotic sensitivity testing was done on all isolates and the resulting profile showed susceptibility reactions of 18 (50.5%), 15 (47.8%), 13 (41.6%), 13 (41.6%) and 13 (41.6%) for nitrofurantoin, ceftriaxone (rocephine), ciprofloxacin, cefuroxime and ofloxacin (tarivid) respectively. This explains that more than 50.0% of the bacterial agents implicated in urinary tract infection in this study were sensitive to nitrofurantoin (Table 4). This is surprising and cheering at the same time. Surprising because, the drug due to its cheapness and availability, ought to be prone to abuse with the attendant development of resistance genes by pathogens to it. It is cheering because patients who go down with UTI can afford it if prescribed. Its use and administration, however, should be closely monitored in order to avoid any possible abuse owing to its accessibility, availability and cheapness. This finding, is however similar to the report of Oluremi et al. (2011) which stated 100.0% sensitivity of *Staph. aureus* to nitrofurantoin.

Less than 50.0% of the uropathogens implicated in this study were sensitive to the third generation cephalosporin–ceftriaxone (rocephine). The next drugs were ciprofloxacin (41.6%), ofloxacin (41.6%) and second generation cephalosporin – cefuroxime (41.6%). The moderate sensitivity of 41.6% for each of the fluoroquinolones (ie, ciprofloxacin and ofloxacin) in this study is low compared with 71.4% and 50.0% for both respectively recorded by Oluremi et al. (2011). This susceptibility level is also low when related to the reports of other workers (Ehinmidu, 2003; Adeleke et al., 2005; Shigemura et al., 2005; Al Sweih, 2005; Mordi & Erah, 2006; Idowu & Odetola, 2007; Mbata, 2007; Saffer et al., 2008; Kolawole, 2009; Omigie et al., 2009).

A much higher sensitivity of the fluoroquinolones and the cephalosporins used in this work was expected. The moderate sensitivity noted and recorded however, was worrisome moreso, as these drugs are expensive and are not readily available for abuse. In a similar study, Nakhjavani et al. (2006) reported that the widespread use of fluoroquinolones in medical centres is a possible cause of high level resistance to fluoroquinolones in UTI patients. True as this may be, it was an intractable task trying to fathom the reason for the high resistance recorded for ceftazidime (85.7%) and cefixime (85.3%) which are cephalosporins that may not be readily available sometimes. Nwadioha et al. (2010) surprisingly recorded a high sensitivity (80.0%) and above of UTI bacterial agents with ceftriaxone, ceftazidime and ciprofloxacin. It is recommended that other workers should point their research telescope in this direction with a view to solving this enigma.

Understandably, gentamicin (an aminoglycoside) and ampicillin (a synthetic penicillin) recorded 79.6% and 71.3% resistance rates respectively. This may be due to their widespread use in the hospitals and healthcare centres (Nakhjavani et al., 2006). Resistance of UTI pathogens to commonly used antibiotics may not be unconnected to their frequent prescription in hospitals, their easy availability in the community without prescription and their low cost which make them subject to abuse (Abubakar, 2009).

Augmentin had a sensitivity of 22.7% and this is disturbing considering its usefulness in treatment of UTI and other infections. According to Oluremi et al. (2011), the total or complete resistance of augmentin is worrisome as it may have lost its value in the treatment of UTI.

Whereas *E.coli* and *Enterobacter* spp resisted three antibiotics each, *Proteus mirabilis* and *Providencia* spp were resistant to four drugs each while *Staph. aureus* and *Pseudomonas aeruginosa* were resistant to five and above six antibiotics respectively in this study (Table 5). A pathogen is multidrug resistant (MDR) when it is resistant to three or more antibiotics at any given time (Jan et al., 2004). Multidrug *Staph aureus* and *Enterococcus* spp have been widely reported in some studies (Gales, 2000; Aiyegoro et al., 2007; Abubakar, 2009). High prevalence of multiple antibiotic resistant strains is a possible indication that very large population of bacterial isolates has been exposed to several antibiotics (Oluremi et al., 2011). The implication of this finding is that most of these pathogens are excretable through urine to the environment. Persons in the Benin City metropolis who live around Ikpoba River and around other rivers who do not have access to bore-hole or pipe-borne water, may be tempted to drink from such rivers into which UTI patients resident around such areas may urinate. The outcome is that unsuspecting residents may drink such MDR pathogens into their systems with its attendant devastating health consequences.

In conclusion, to reduce community antibiotic resistance of commonly occurring pathogens in different populations particularly with regard to UTI, the Healthcare policy of antibiotics prophylaxis should be reviewed in order to address emergence of resistance genes as a consequence. Such a review should be directed at regular reporting of sensitivity patterns of pathogens of UTI and other diseases before commencement of therapy, the use and type of which should depend on its toxicity, affordability and effectiveness.

Use of combination of drugs for synergistic effect should be encouraged. As a consequence, the emergence of resistant strains within any population could be checkmated and patients will be better managed holistically. As a consequence also, the dissemination of MDR pathogens would be curtailed. Government of the day should endeavour to provide pipe-borne water to all areas within its jurisdiction. Health care delivery agencies should encourage the people to imbibe improved personal hygiene techniques through extensive health education. Landlords must be made to provide standard and clean toilet facilities for their tenants and erring landlords should be sanctioned.

## 5. Conclusion

In order to reduce community antibiotic resistance of commonly occurring pathogens in different populations particularly with regard to UTI, the Healthcare policy of antibiotics prophylaxis should be reviewed in order to address emergence of resistance genes as a consequence. Such a review should be directed at regular reporting of sensitivity patterns of pathogens of UTI and other diseases before commencement of therapy, the use and type of which should depend on its toxicity, affordability and effectiveness. Use of combination of drugs for synergistic effect should be encouraged. As a consequence, the emergence of resistant strains within any population could be checkmated and patients will be better managed holistically. As a consequence also, the dissemination of MDR pathogens would be curtailed. Government of the day should endeavour to provide pipe-borne water to all areas within its jurisdiction. Health care delivery agencies should encourage the people to imbibe improved personal hygiene techniques through extensive health education. Landlords must be made to provide standard and clean toilet facilities for their tenants and erring landlords should be sanctioned.
